# A Need for Improvement in the Definition of Resistant Arterial Hypertension

**DOI:** 10.3390/medicina59040803

**Published:** 2023-04-20

**Authors:** Goran Koracevic, Sladjana Micic, Milovan Stojanovic, Marija Zdravkovic

**Affiliations:** 1 Department for Cardiovascular Diseases, Clinical Center Nis, 18000 Nis, Serbia; gkoracevic@yahoo.com; 2 Faculty of Medicine, University of Nis, 18000 Nis, Serbia; 3 Clinic for Nephrology, University Clinical Center Nis, 18000 Nis, Serbia; sladjana.micic@live.com; 4 Institute for Treatment and Rehabilitation Niska Banja, 18000 Nis, Serbia; 5 University Hospital Medical Center Bezanijska Kosa, 11000 Belgrade, Serbia; direktor@bkosa.edu.rs

**Keywords:** blood pressure, arterial hypertension, resistant hypertension

## Abstract

With the medical and social importance of resistant arterial hypertension (HTN) in mind, we had three goals in this paper: to study the definitions of resistant HTN in the guidelines on the topic, to analyze them, and to suggest some improvements. We found (at least) eleven insufficiencies in the definition of resistant HTN: (1) different blood pressure (BP) values are used for diagnoses; (2) the number of BP measurements is not specified; (3) the *time-frame for the definition* is not obtained; (4) it fails to provide *normal* or *target* or *controlled* BP values; (5) secondary HTN is not currently defined as true resistant HTN, but as apparently treatment-resistant HTN; (6) the definition usually directly incorporates BP cut-offs for systolic BP (sBP) and diastolic BP (dBP) making the diagnosis temporary; (7) *stress is not included* in the exclusion strategy for resistant HTN; (8) there is potentially a need to introduce a category of *recovered* resistant HTN; (9) to what *degree do healthy lifestyle measures have to be fulfilled* to consider it as sufficient to change the diagnosis from “apparent treatment-resistant HTN” to the “resistant HTN”; (10) sBP values *normal-for-the-age* for 61 and 81 year old patients in some guidelines fulfill the criterion for resistant HTN; (11) it probably ought to read “In the absence of contraindications and compelling indications…” in the others. We believe that it is better to use the phrase “above the target BP” for the definition of (treatment) resistant HTN, because the whole story of resistant HTN is related to non-responders to antihypertensive treatment. Therefore, as we *treat to target* and not to normal values, it is appropriate to define resistant HTN as an insufficiency to reach the target BP values. Moreover, the definition of (treatment) resistant HTN should not be universal for every patient with HTN, but it should be age-related: (treatment) resistant HTN is elevated BP ***over*** the target/normal BP values. Using this modification, there will be no need to automatically change the definition of resistant HTN when we change the BP targets in the future.

## 1. Introduction

In the 2020 International Society of Hypertension’s *Global Hypertension Practice Guidelines*, resistant arterial hypertension (HTN) is defined as blood pressure (BP) measured at over 140/90 mmHg in the doctor’s office in patients who receive ≥3 antihypertensive drugs (diuretics included) in doses that are either optimal or maximally tolerated following the exclusion of pseudoresistance, which includes an improper method for BP measurement that ignores the white-coat effect, drug-induced HTN, and secondary HTN, as well as mistakes regarding antihypertensive therapy either by a physician (suboptimal antihypertensive drugs choices) or by a patient (treatment nonadherence) [1]. HTN without pseudoresistance is named “true resistant HTN” and with pseudoresistance is called “apparently treatment-resistant HTN” [2].

Resistant HTN is very important due to both its high prevalence and risk [2,3]. As for the prevalence of true resistant HTN, the estimation of the *Global Hypertension Practice Guidelines* is approximately 10% of the hypertensive population [2]. Numerous authors quote 10–20% without referring to true or apparently treatment-resistant HTN [2,4,5]. Indeed, with a lower cut-off (130/80 mmHg) as recommended by American Heart Association’s (AHA) 2018 guidelines [6], the prevalence of resistant HTN is higher (20%) [7]. Another respective source—Hypertension Canada’s 2020 *Evidence Review and Guidelines* cite the prevalence of resistant HTN as 10–30% [3]. On the other hand, the anticipated prevalence of true resistant HTN in another paper was 2% [8]. Finally, a large meta-analysis, which included 91 epidemiological studies and 3,207,911 patients with HTN, demonstrated that the prevalence of true resistant HTN and of pseudoresistant HTN was about 10% each [9].

We noticed several unclear and controversial aspects of resistant HTN in the contemporary publications. It is disappointing to have both 130 and 140 mmHg as cut-offs for resistant HTN. The difference in systolic BP (sBP) of 10 mmHg translates to a huge prognostic difference during the follow-up. For example, 10 mmHg decrease in sBP under the antihypertensive treatment can be expected to diminish the risk of cardiovascular events by 20%, according to a recently published meta-analysis that included 344,716 participants [10]. Moreover, an earlier meta-analysis of >1,000,000 patients and more than 12,700,000 person-years demonstrated at least a twofold difference in death rates of ischemic heart disease, stroke, and other vascular diseases for each 20 mmHg in usual sBP [11]. Indeed, not only the decisions regarding individual patients but also the global prevalence of resistant HTN depends greatly on the cut-off; with the lesser cut-off of 130 mmHg for sBP, the prevalence of resistant HTN is one-fifth higher (as compared with 140 mmHg) [7]. If we assume HTN prevalence is >1 billion patients [12,13,14,15] and the prevalence of resistant HTN at least 10% [2], then these different cut-offs for resistant HTN concerns >100 million patients worldwide.

The rationale for this paper is the following: The most important aspects of resistant HTN are covered by the current definition(s), e.g., according to the principles for a good definition recommended in the paper of Oliver and Perry [16]. Nonetheless, the importance of HTN in general (and particularly of resistant HTN) imposes a need for improvements, because even the smallest development could provide significant benefits globally. The immediate motive consists of several inconsistencies regarding resistant HTN that we have observed. Knowing that approximately 1.4 billion patients have HTN globally [17], 10–20% of them is a population of approximately 140–280 million people across the world with resistant HTN. This 10–20% with resistant HTN have a worse prognosis than the other 80–90% of the HTN population, which is those who are not resistant. This is because of the high risk that is imminent to resistant HTN [1,2,3,7,18]. With the medical and social importance of resistant HTN (stemming from the very high prevalence and prominent risk) in mind, we had three goals in this paper: to study the definitions of resistant HTN in the guidelines on the topic, to analyze them, and to suggest some potential improvements.

## 2. Materials and Methods

**Figure 1:**
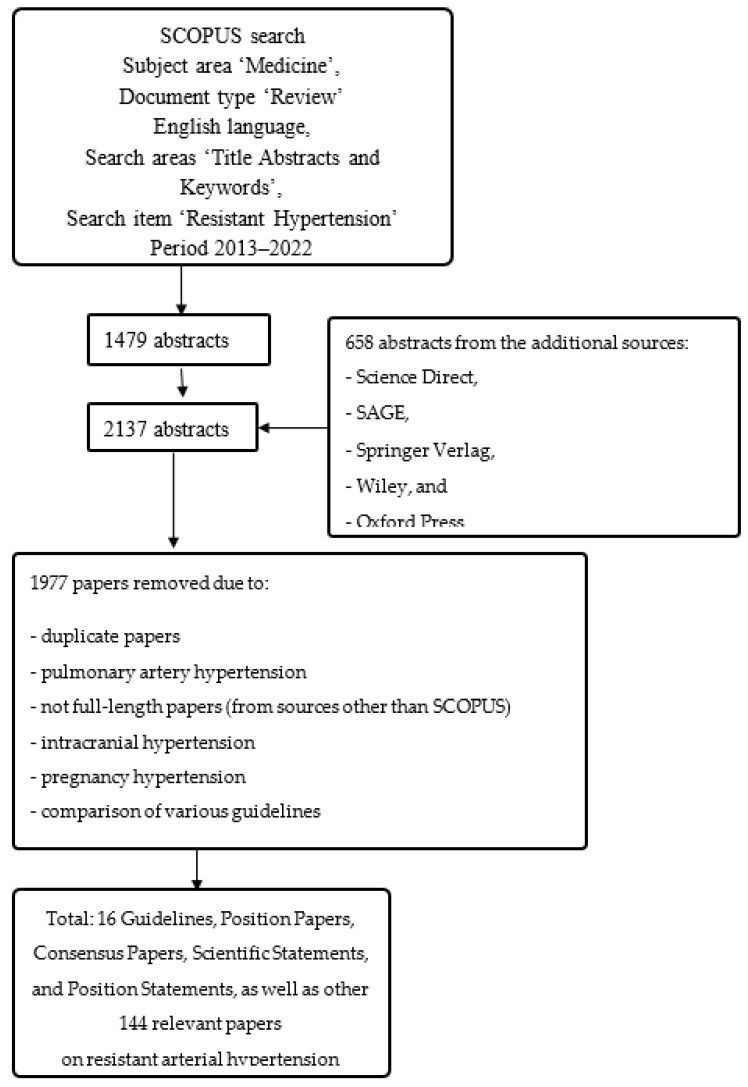
A flow chart for the search for resistant HTN, mostly in HTN guidelines. We performed a narrative review aiming to analyze the differences and shortcomings in the available papers, mostly using guidelines.

The search began on SCOPUS (Figure 1). The search term was ‘resistant hypertension’. The time window used was 2013–2022 and the language was English. Articles and reviews in the subject area of ‘Medicine’ were looked for. The search areas were ‘Title, Abstracts and Keywords’. In the first place titles were analyzed for relevance resistant hypertension, and then the abstracts were read. In total, 2137 abstracts were retrieved on 21 December 2022. If the abstract was relevant, full-length papers were retrieved and studied. If needed, the “snowball search” was performed in Science Direct, SAGE, Wiley, Springer, and Oxford Press. The articles were not subjected to quality assessment, because the aim was not to evaluate the key documents we analyzed (i.e., Guidelines), neither by the National Guideline Clearinghouse Extent of Adherence to Trustworthy Standards (NEATS) Instrument nor by the Appraisal of Guidelines for Research and Evaluation (AGREE-II); we considered Guidelines being of utmost importance due to their direct powerful influence on clinical practice.

## 3. Results

After reviewing the literature, we made the following synthesis. The definition of resistant HTN is suboptimal due to at least 11 reasons (Table 1).

The first unsolved issue is the BP cut-off to diagnose resistant HTN, because 4 different cut-offs were used in the previous 5 years: >140/90 mmHg [1], ≥140/90 mmHg [4], >130/80 mmHg [11], ≥130/80 [19,20], and various cut-offs (without a single cut-off) [3]. The last modality also has clear rationale and it needs to be applied in clinical practice (because methods for BP measurements and therapeutic targets are not universal) [3].

The second problem is that the identical systolic BP (sBP) value (e.g., 145 mmHg on the adequate triple antihypertensive therapy), which is normal-for-the-age for a 61 year old patient [21], and also the normal-for-the-age sBP for an 81 year old [22,23], fulfil the criterion for resistant HTN both [1,6,22,24,25,26]. Indeed, these two categories (“normal” BP and “resistant” HTN) are exclusive of each other.

The third possible shortcoming is that the number of BP measurements is rarely specified. For example, are the criteria for resistant HTN fulfilled with a single measurement in the office (while on proper three antihypertensive drugs treatment) or is there a need for several measurements? Keeping the variations of BP measurement results when taken in a doctor’s office in mind, it is reasonable to have several measurements before the diagnosis of apparent treatment-resistant HTN is made (to diminish the probability of measurement error). This view is also in concordance with the recommendation to perform several BP measurements in order to make the diagnosis of HTN for the first time (except for severe BP elevations) [22].

Therefore, we do not have guidelines’ recommendation with regards to how many BP measurements should be obtained. Indeed, longer observation periods mean that more measurements are possible (and the other way round). There is an opinion to start from; Fay and Cohen recommended the following: 2 readings in the morning (prior to antihypertensive drugs) and an additional 2 in the evening (prior to sleep); measurements should be done with minimum 1 min pause [27]. Undoubtedly, the preferred technique for BP measurements is to be considered when recommending the number of BP readings. Generally, ambulatory blood pressure monitoring (ABPM) is needed (to exclude white-coat HTN and confirm resistant HTN), but this is not available around the globe.

Related to the number of measurements is the forth possible shortcoming—that the time-frame for the definition is not obtained, i.e., how long BP should be uncontrolled to diagnose resistant HTN. Is this a single measurement or 15 days or 1 month? Generally, the recommendation is absent or not quite precise. Many patients have increased BP in the doctor’s office at least partially due to temporary stress (including the white-coat effect). A single BP measurement is probably not enough to label HTN ‘resistant’; therefore, repeated BP measurements are warranted for some time. Moreover, antihypertensive drugs need time to exert full effect.

Furthermore, it seems reasonable to allow patients one or several weeks to improve healthy lifestyle habits (such as to control excessive salt, alcohol, and tobacco consumption) and allow the antihypertensive drugs to show full effect, and then reassess the presence of the apparent treatment-resistant HTN (if secondary HTN and other required elements are not excluded) or true resistant HTN (if they are). There is a general suggestion that resistant HTN implies failure to reach BP targets during the next month following the start of an adequate drug regimen [28]. We were only able to find one paper that provided a recommendation: a minimum of 3 days (and optimally ≥7 days) with 4 measurements per day, preferably during consecutive days [27].

The fifth problem is the unresolved issue of whether or not to diagnose resistant HTN if triple optimal treatment fails to provide normal or target or controlled BP values. For example, in the ESC/ESH guidelines, normal sBP is <140 mmHg, target sBP is 130 mmHg (for the majority of patients), and 135 mmHg means normal sBP has been achieved while failing to meet the target sBP level [22]. BP values higher than target are also cited as criterion for resistant HTN [2,3,29,30,31]. “Uncontrolled” BP values are used to define resistant HTN in the Canadian and American guidelines [6,23,32].

The sixth shortcoming is the absence of a criterion describing the degree in which healthy lifestyle measures need to be fulfilled to allow us to say it is sufficient to change the diagnosis from “apparent treatment-resistant HTN” to the “resistant HTN”. For example, how should we check patients’ salt intake in everyday practice: 24-h urinary sodium excretion, food frequency questionnaire, spot urine collection, or another tool [33,37,38,39,40]? How realistic are self-reports? [33,38]. As a rule, it is not enough to simply remove the saltshaker from the table, and it is difficult to measure quantities of food consumed and the grams of salt that the food contains [23,33,35].

How many patients succeed in complete healthy lifestyle adoption? Numbers are low [41,42,43,44,45], and the question remains—for how long does a healthy lifestyle need to be maintained [42,46,47,48]? We are aware that drug adherence is disappointingly low (e.g., 20–50%) [49,50,51], and that adopting a healthy lifestyle needs much more effort and time [52]. Lifestyle changes are well-recognized as difficult to perform [53,54], e.g., quitting smoking [55]. Therefore, the number of true resistant HTN patients may be lower (e.g., 2%) [8] than what is reported [5,9].

Is it a pre-requisite for the diagnosis of true resistant HTN to exclude all mistakes in the lifestyle? It is also important to underline the need for complete/sufficient compliance with a healthy lifestyle because it is well recognized that excess food or mistakes in diet (salty food and salty drinks, such as mineral water) and insufficient physical activity can elevate BP and contribute to resistant HTN, as does excessive alcohol and nicotine abuse.

The seventh small insufficiency is the absence of a place/name for the category of patients who adopt a healthy lifestyle completely and no longer need ≥3 antihypertensive drugs. Is there a need to introduce also a category for recovered resistant HTN? There are patients with the apparent treatment-resistant HTN (or even true resistant HTN) who (in addition to other lifestyle improvements) lose enough weight to obtain very good BP control with a drug or two (instead of previously used 3 or 4). Therefore, they no longer have resistant HTN, and instead have recovered resistant HTN. This group would be an absolute analogy with the inclusion of “the recovered ejection fraction (EF)” subgroup in the definition of heart failure [56,57]. Recovered resistant HTN is an important category, and Tsioufis et al. found that 10% of initially resistant HTN improved enough to be included in this category [36].

The eight potential insufficiency is that to diagnose true resistant HTN, a physician needs to exclude secondary hypertension, poor antihypertensive drug adherence, as well as several types of unhealthy behavior, such as excessive consumption of salt, alcohol, or drugs that increase BP. This is a commonplace, well-accepted strategy in numerous guidelines and papers on the topic [2,8,27]. It is difficult to find a reason why stress is not included in this strategy despite the known effects of stress upon HTN genesis and worsening [58,59,60,61]. We all are aware of how much stress worsens HTN regulation—intensive stressors (e.g., death of close relative or spouse) and/or excessive reaction to sometimes an ordinary level of stress may not only cause difficult-to-treat HTN, but also a hypertensive crisis. Moreover, some degree of stress is typical for modern times. Indeed, it is not easy to measure it in everyday busy practice, but other measurements of lifestyles are impractical, too.

The ninth limitation may be the absence of a logical recommendation—the guidelines probably ought to read “In the absence of a compelling indication…”. Resistant HTN is a more severe type of HTN (as compared to non-resistant) with a worse prognosis, and the hypertension-mediated organ damages (HMODs) are/will be obvious in many patients. Therefore, it makes sense to expect many HMODs in patients with resistant HTN [62]. Moreover, one or a few side effects or HMODS are very likely in patients with resistant HTN [22,31,63,64].

Here we come to the point: if co-morbidities and HMODs are reasonably expected in resistant HTN [65,66], they would impact the drug choice for those patients (with resistant HTN) [2,28], because they represent the compelling indications recognized in many guidelines [63]. For example, if there is a tendency toward ischemic heart disease (IHD) or if it is already diagnosed, the optimal 3 drugs for uncomplicated HTN will have to include recommended treatments for IHD, such as beta blocker (BB), calcium channel blocker (CCB), and renin-angiotensin-aldosterone (RAAS) blocker [22].

Compelling indications are usually co-morbidities [67]. Moreover, compelling indications for some antihypertensive drugs are not rare in apparently treatment-resistant HTN, because are associated with a higher prevalence of HMODs and co-morbidities [4,68,69]. For example, if a patient has apparently treatment-resistant HTN and chronic coronary syndrome (CCS), the guideline-recommended treatment would be BB (in addition to dihydropyridine CCB and RAAS blocker) but not diuretics [1,22,28,70,71,72]. To sum up, it probably ought to read “In the absence of contraindications and compelling indications…”

The tenth potential shortcoming with the definition of HTN is that it usually directly incorporates BP cut-offs for sBP and diastolic BP (dBP). It is excellent solution from a practical viewpoint for some time, but not for a long time because:

(A) The definitions of normal BP are geographically unequal—they are not the same in Europe and USA [6].
(B) BP cut-offs to define normal BP have changed over time, and probably will continue to do so. If we omit exact BP cut-offs directly in the definition, it will stay the same over time (e.g., failure to obtain normal/target/controlled BP despite optimal (as suggested by the guidelines), triple antihypertensive drugs treatment, and non-medicament antihypertensive treatment) and the essence of the definition would be easier to interpret over time. The only thing that probably will change are the cut-offs for normal/target BP, which ought to be stated in the next sentence following the definition of the BP cut-off.
(C) Cut-offs for resistant HTN depend on the method used for the measurement [73].

The eleventh potential insufficiency is that increased BP despite triple optimal antihypertensive therapy due to secondary HTN is not currently defined as “true resistant HTN”, but as “apparently therapy resistant HTN” [1]. We believe it is clinically resistant, no matter if and when we will find the cause (e. g., renal artery stenosis) and confirm it is a secondary HTN. There is a possibility that BP in an individual patient with resistant HTN might be one day much improved (by treating the cause of secondary HTN, i.e., by curing secondary HTN); the possibility that this HTN might stop being resistant in the future does not mean that it is not resistant now.

## 4. Discussion

The main finding of this narrative review is a list of shortcomings of the current approach to resistant HTN. Resistant HTN is a prevalent [2,4,5,74] and high-risk [2,3,18] subset of HTN, and has a population that exceeds 1/10 of 1.4 million HTN patients worldwide [17]. Therefore, even a small improvement regarding resistant HTN would have significant clinical consequences. We believe it is better to use the phrase “above the target BP” for the definition of (treatment) resistant HTN, because the whole story of resistant HTN is related to non-responders to antihypertensive treatment. Therefore, as we aim to treat to target and not to normal values, it is appropriate to define resistant HTN as the insufficiency to reach the target BP values.

Moreover, we suggest a modification of the definition of (treatment) resistant HTN, which should not be universal for every patient with HTN, but age-related: (treatment) resistant HTN is elevated BP over the target/normal BP values; ≥140/90 mmHg for patients below the age of 80 years, and 150/90 mmHg for patients older than 80 years, according to European guidelines [22]. Using this modification, there will be no need to automatically change the definition of resistant HTN when we change the BP targets in the future. 

The purpose of the current article is to focus scientific attention on the possible shortcomings of resistant HTN definitions, which is the first step toward potential improvements. The solutions to the insufficiencies observed are far above the scope of this narrative review. Therefore, Table 2 below is only meant to be a provisory starting point to begin working with and is by no means the definitive answer.

It is important to have complete but clear definitions, operative enough to be used in everyday clinical practice for the diagnostic purposes. Such definitions by experts in the international societies should become officially universal (such as definitions for myocardial infarction and heart failure, which were produced by world societies/task forces) in order to be used globally. Proper definitions improve our individual therapeutic approaches, e.g., which combination of antihypertensive drugs can be expected to be optimal for a particular patient with resistant HTN. Moreover, guideline- directed medical treatment is recognized as very useful worldwide. It is particularly true for very prevalent diseases, such as HTN.

## 5. Conclusions

Resistant HTN represents one of the highest-risk groups of HTN, which is the main cause of morbidity and mortality in the world. Several insufficiencies and inconsistencies are observed in the definitions and approaches to resistant HTN, and solving them can improve the current approach to this very important problem. The first step is to be aware of the shortcomings, and the solution does not require extensive trials, but rather experts’ consensus.

## Figures and Tables

**Table 1:** Insufficiencies in the definition of resistant HTN.

Remarks (Insufficiencies in the Definition of Resistant HTN)	References
1. Different BP cut-offs to diagnose resistant HTN are used.	[1,3,4,18,19,20]
2. sBP values *normal-for-the-age* for 61 and 81 year old patients in some guidelines fulfill the criterion for resistant HTN.	[6,21,22,23,24,25,26]
3. The number of BP measurements is not specified.	[22,27]
4. The *time-frame for the definition* is not obtained.	[27,28]
5. *Normal* or *target* or *controlled* BP values have not been provided.	[2,3,6,22,23,29,30,31,32]
6. To what *degree do healthy lifestyle measures need to be fulfilled* to consider it as sufficient to change the diagnosis from “apparent treatment-resistant HTN” to “resistant HTN”?	[33,34,35]
7. Is there a need to introduce a category of *recovered* resistant HTN?	[36]
8. *Stress is not included* in the exclusion strategy for resistant HTN.	[2]
9. It probably ought to read “In the absence of contraindications and compelling indications…”.	n/a
10. The definition usually directly incorporates BP cut-offs for sBP and dBP (which is not an ideal solution from a practical viewpoint).	[1]
11. Secondary HTN is not currently defined as true resistant HTN, but instead as treatment-resistant HTN.	[1]

Legend: BP—blood pressure; HTN—arterial hypertension; sBP—systolic BP; dBP—diastolic BP.

**Table 2:** Suggestions for the improvement of current definition, as a starting point, to be considered by experts in the field in hypertension societies.

Remarks (Insufficiencies in the Definition of Resistant HTN)	Suggestions for Improvement
1. Different BP cut-offs to diagnose resistant HTN are used.	An international consensus could solve this by setting the sBP cut-off ought to either 130 or 140 mmHg.
2. sBP values considered *normal-for-the-age* for 61 and 81 year old patients fulfill the criterion for resistant HTN in some guidelines.	This shortcoming may be improved by paying more attention to not mix normal and abnormal BP ranges; it may be stated in the national guidelines that (in the case of differences) international guidelines should have priority in clinical consideration.
3. The number of BP measurements is not specified.	A single BP measurement following the introduction of triple antihypertensive treatment is clearly not enough; dose up-titration needs to be applied. Therefore, at least 3 additional doctor’s office BP measurements are likely needed.
4. The *time-frame for the definition* is not obtained.	This is clearly a point where consensus is needed; but it depends on several factors. Possibly, for most patients, this time-frame might be a month.
5. Normal, target, and controlled BP values are not provided.	It seems that a failure to achieve *target* rather than normal BP values can be considered *treatment*-resistant HTN, because the aim of *treatment* is to provide *target* BP.
6. What is the degree to which healthy lifestyle measures need to be fulfilled to consider it as sufficient to change the diagnosis from “apparent treatment-resistant HTN” to “resistant HTN”?	This is important, but difficult to define and it obviously needs scientific work and consensus.
7. Is there a need to introduce a category for *recovered* resistant HTN?	Although this category is rarely mentioned, the answer seems to be positive.
8. Stress is not included in the exclusion strategy for resistant HTN.	Stress is not easy to predict and measure in routine clinical practice, but it should be considered in each patient, prevented/treated within the limits of reality, and evaluated similarly as other risk factors, e.g., increased salt intake.
9. It probably ought to read “In the absence of contraindications and compelling indications…”.	The answer is positive, because of the high number of various comorbidities in patients with resistant HTN; they represent contraindications for some and compelling indications for the other antihypertensive drugs.
10. The definition usually directly incorporates BP cut-offs for sBP and dBP (which is not an ideal solution from a practical viewpoint for a long time).	This can be solved by using the phrase “above the target BP” possibly with the additional explanation “currently above this cut-off”.
11. Secondary HTN is not currently defined as true resistant HTN, but as apparently treatment-resistant HTN.	Secondary HTN might be considered true resistant HTN, because it usually cannot be controlled by adopting a healthy lifestyle and following other recommended measures.
